# Oral Kaposi sarcoma following cord blood transplantation

**DOI:** 10.1002/jha2.387

**Published:** 2022-01-24

**Authors:** Nobuhiko Imahashi, Mikiko Arakawa, Akari Iwakoshi, Mikiko Mori, Hirokazu Nagai, Hiroatsu Iida

**Affiliations:** ^1^ Department of Hematology National Hospital Organization Nagoya Medical Center Nagoya Japan; ^2^ Department of Oral Surgery National Hospital Organization Nagoya Medical Center Nagoya Japan; ^3^ Department of Pathology National Hospital Organization Nagoya Medical Center Nagoya Japan; ^4^ Department of Infectious Disease National Hospital Organization Nagoya Medical Center Nagoya Japan

**Keywords:** cord blood transplantation, immunodeficiency, Kaposi sarcoma

A 50‐year‐old man developed acute myeloid leukemia while receiving antiretroviral therapy for human immunodeficiency virus (HIV) infection. Following three courses of chemotherapy, he received cord blood transplantation (CBT) in the first complete remission. The patient developed mild skin acute graft‐versus‐host disease after CBT. In addition, he developed organizing pneumonia 1 month after CBT, which responded to steroid therapy, but repeatedly relapsed during steroid tapering. Thus, he had been receiving prolonged immunosuppressive therapy. Thirteen months after CBT, bluish‐red elevated lesions developed on the hard palate of the oral cavity (Figure 1A). Biopsy samples obtained from the palate lesions confirmed the diagnosis of Kaposi sarcoma (KS). His HIV viral load was undetectable, and there were no signs of leukemia relapse. To improve the immunosuppression status, immunosuppressive therapy was attenuated. This led to the increase in CD4^+^ count from 0.26 × 10^9^/L to 0.68 × 10^9^/L and regression of the KS (Figure 1B, 18 months after CBT). However, the patient succumbed to heart and kidney failure 18 months after CBT. Despite its rarity, KS should be considered as a differential diagnosis when hematopoietic stem cell transplantation recipients develop bluish‐red or purple lesions.

**FIGURE 1:**
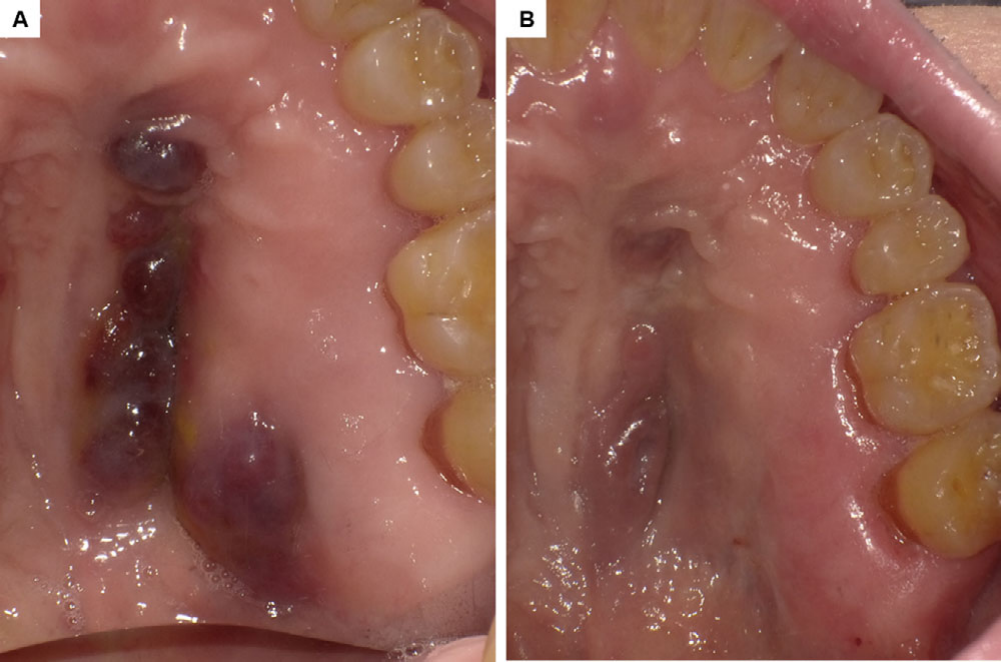
Oral Kaposi sarcoma that developed after CBT. (A) At the time of diagnosis (13 months after CBT). (B) After attenuation of immunosuppressive therapy (18 months after CBT)

## CONFLICT OF INTEREST

The authors declare no conflict of interest.

## AUTHOR CONTRIBUTIONS

N.I., M.A., A.I., M.M, H.N., and H.I. were involved in the management of the patient. N.I., H.N., and H.I. wrote the manuscript. N.I., M.A., and A.I. prepared the figure. All authors reviewed and approved the manuscript.

## CONSENT STATEMENT

Informed consent was obtained from the patient.

